# Occurrence of changes in the auditory evoked potentials of smokers: systematic review of the literature

**DOI:** 10.1590/2317-1782/20232021273en

**Published:** 2023-08-04

**Authors:** Dayane Stephanie Potgurski, Georgea Espindola Ribeiro, Daniela Polo Camargo da Silva

**Affiliations:** 1 Departamento de Fonoaudiologia, Universidade Federal de Santa Catarina - UFSC - Florianópolis (SC), Brasil.; 2 Hospital das Clínicas da Faculdade de Medicina da Universidade de São Paulo - HCFMUSP - São Paulo (SP), Brasil.

**Keywords:** Smokers, Electrophysiology, Hearing, Cochlear Nerve, Auditory Evoked Potentials

## Abstract

**Purpose:**

To verify the occurrence of abnormal auditory evoked potentials (AEP) tests in adult smokers.

**Research strategies:**

Systematic review of the literature according to the PRISMA guidelines, to answer the question: “Are there any changes in the AEP results in adult smokers?”, PECOS strategy. Research carried out on PubMed, Embase, CINAHL, LIVIVO, Scopus, Web of Science, LILACS and Scielo databases. Additional search of gray literature: Google Scholar and ProQuest hand searching of reference lists of the included studies.

**Selection criteria:**

Cross-sectional studies were selected, without restriction on the year of publication and language.

**Data analysis:**

First, the titles and abstracts of all the studies were analyzed, followed by the full reading of the eligible studies.

**Results:**

898 articles were collected, after the duplicate studies were removed and after blind analysis by three researchers, 8 studies of the observational type were selected. Most studies have found an association between active smoking and changes in electrophysiological tests.

**Conclusion:**

Normal hearing adult smokers present alterations in short and long AEP. In the auditory brainstem response, the main altered components were the increase in waves latencies of I and III and in the interpeaks I - III and III - V, as well as a decrease in the amplitude of the waves. In Mismatch Negativity, there was a significant increase in wave amplitude and latency. In the long latency potential, P300, there was an increase in latencies and decreased amplitudes in the components N1 (in Fz) and P3.

## INTRODUCTION

Tobacco consumption is considered one of the major risk factors that cause morbidity and mortality worldwide and it is known that its consumption is main nicotine release way, which is highly absorbed into the bloodstream and can compromise different structures of the organism, producing several harmful effects, such as heart disease, stroke, chronic obstructive pulmonary disease, as well as cancer and cognitive impairment, among others^(1,2)^.

Tobacco also causes decreased cell oxygenation, vascular blockage, changes in blood viscosity, formation of atherosclerotic plaque, and decreased oxygen supply, which can lead to impaired blood supply, including to the auditory pathways^(1-3)^.

Therefore, the auditory system can be exposed to harmful influences from this adverse event and, when it comes to ototoxicity, its effects can be transient or permanent, depending on which structures were affected or the characteristics of the exposure^(4)^. Also, as any type of hearing impairment can lead to a worsening of the individual's quality of life, many authors have explored the association between tobacco use and its effect on hearing over the years^(5)^.

A recent 8-year cohort study verified the prospective association of tobacco use, intensity, and smoking cessation with the risk of hearing loss, which included 50,195 participants, aged between 20 and 64 years and without hearing loss at the beginning of the study. Pure-tone threshold audiometry was performed annually, and during monitoring, 3,532 individuals developed high-frequency hearing loss and 1,575 developed low-frequency hearing loss. The conclusion was that smoking is associated with an increased risk of hearing loss, especially at high frequencies, in a dose-response manner. The excessive risk of hearing loss associated with smoking disappeared in a relatively short period after smoking cessation^(5)^.

In addition to the occurrence of sensorineural hearing loss at high frequencies, a study also observed the presence of a high number of smokers with tube dysfunction, which increases the incidence of middle ear diseases, as it brings nonspecific symptoms characterized by ear fullness and difficulty equalizing the middle ear^(6)^.

Furthermore, nicotine can be transported to receptors in the central nervous system and may involve both peripheral and central auditory structures^(1,7)^. It is also known that the degeneration of the function of the nervous system happens mostly in a rostrocaudal manner, that is, it starts with the cortex, passing through the subcortical regions until reaching the brainstem^(8)^. Thus, the investigation of the nicotine effect on the central auditory nervous system (CANS) has also been investigated by means of the auditory evoked potential (AEP).

The AEP is a set of methods that evaluate the electrobiological activity along the auditory system, from the inner ear to the cerebral cortex. Thus, the application of these tests allows the investigation of hearing neurophysiological conditions^(9)^.

The AEPs can be divided into three types. The brainstem auditory evoked potential (BAEP), considered a short-latency potential, stands out; it appears in an interval of approximately 10 ms after stimulation, which allows the neurophysiological analysis of the auditory pathway, from the inner ear to the high brainstem^(10)^. The middle-latency auditory evoked potential, which appears approximately 80 ms after stimulation, originates in the primary auditory cortex - more specifically from the nuclei and auditory pathways to the level of the thalamus-cortical region and primary auditory cortex^(11)^; finally, the long-latency auditory evoked potential, which appears around 100 to 700 ms after stimulation, reflecting activities of the auditory pathway in the regions of the thalamus and auditory cortex, providing information about the CANS functioning^(12)^.

Thus, sensorineural hearing loss at high frequencies of cochlear origin compromises the morphology of AEP waves, as well as retrocochlear disorders. In this sense, studies indicate that there are alterations in the parameters of AEP test records in smokers when compared to non-smokers, such as increased latencies and decreased response amplitudes, even in the absence of increased auditory thresholds, and these findings may somehow influence the correct processing of acoustic information^(7,13,14)^.

Several anatomical sites, responsible for producing the neuroelectric activity of the auditory pathway in response to acoustic stimulation, may behave differently in smokers, which makes it necessary to assess what evidence is available in the literature that proves the existence or not of that association.

In view of the above, studies have pointed to the need to carry out additional tests for basic audiological assessment in order to investigate the extent of the injury caused by constant tobacco exposure, helping in a better understanding of the alterations found, besides detailing the types of alterations that smoking causes to the CANS^(2,7,13,14)^.

Thus, the AEPs analysis proves to be useful in the differential diagnosis of sensorineural hearing loss, bringing important pieces of information that can indicate objectively if the lesion is located at the cochlear and/or retrocochlear level, as well as showing early changes in the sites that generate the neural response, before changes are detected in the basic audiological assessment^(2,7,13,14)^.

## PURPOSE

This study aimed to verify the occurrence of abnormal auditory evoked potentials (AEP) tests in adult smokers with normal hearing, through a systematic review of the literature.

### Research strategy

This systematic review followed the recommendations of the Preferred Reporting Items for Systematic Reviews and Meta-analyses - PRISMA^(15)^.

Observational studies on adult smokers (aged 18 years and under 60 years) were considered eligible for this systematic review, which aimed to assess the integrity of the auditory pathway by means of the AEP test. There was no restriction on the study publication date. The following studies were excluded: (1) review articles, letters, case studies, and event abstracts; (2) studies on smokers associated with other diseases; (3) studies that included individuals younger than 18 years old or older than 60 years old; (4) studies carried out with smokers with hearing alterations prior to addiction; (5) studies without a control group; (6) studies without the full version available.

The following guiding question was used to conduct the study: “Are there any changes in the AEP results in adult smokers?” The PECOS approach^(16)^ (Patient, Exposure, Comparison, Outcomes, Studies) was used to formulate the guiding question. Thus, in this literature review, PECOS stands for P - population (adults), E - exposure to tobacco; C - tobacco non-exposed adults; O - any change in auditory evoked potential tests, and S - design of included, observational cross-sectional studies (S).

Individual electronic search strategies were developed, using the combination of the following descriptors in Portuguese and English, respectively: “Fumantes,” “Potenciais Evocados Auditivos,” “Eletrofisiologia,”, “Nervo Coclear,” “Smokers”, “Auditory Evoked Potentials,” “Electrophysiology,” “Cochlear Nerve.” In order to encompass certain thematic axes, the Boolean operators “OR” and “AND” were used, according to MESH/DECS, for each of the following databases: PubMed, Embase, CINAHL, LIVIVO, Scopus, Web of Science, LILACS, and Scielo. Additional search of gray literature was made by accessing Google Scholar and ProQuest. Moreover, hand searching of reference lists of the included studies was conducted, as recommended by Greenhalgh and Peacock (2005)^(17)^. EndNote Web®, reference manager software, was used to collect references and delete duplicates. The collection date on the databases was held on March 24, 2021. Studies that answered the research question were selected, without restriction on publication date and language.

### Selection criteria

The selection stage had two phases. In phase one, the titles and abstracts of all identified database citations were screened by two reviewers independently. Studies that did not meet the eligibility criteria were excluded. In phase two, the same two reviewers applied the eligibility criteria to the full text of the studies. A third reviewer was consulted in case of disagreement that was not resolved by a consensus discussion between the two reviewers.

### Data analysis

Two authors collected the necessary information from the selected studies. A third author confirmed the veracity of the information collected by checking the full text of the articles against the information selected by the first two authors. Any controversies in this process were discussed and a consensus was established. The data extracted from the studies were: characteristics of the studies (authors, year of publication, country, type of study), characteristics of the population (sample size, age range of the group studied), characteristic of exposure (smoking characterization), and characteristics of the outcome (type of alteration found in the retrocochlear auditory pathway, type of AEP used, and main findings presented by the studies).

The risk of bias assessment of the selected studies was evaluated using the JBI Critical Appraisal Checklist for Studies Reporting Prevalence Data^(18)^. The first and second authors performed this assessment independently. Any disagreements that arose were resolved with the third author’s help.

For each domain of the tool, one of the following responses was assigned: “Yes,” “No,” “Unclear” or “Not Applicable”^(13)^. Regarding the percentage of “Yes” of each analyzed study, classification was as follows: high risk of bias (> 49%), moderate high risk of bias (50 to 69%), or low high risk of bias (> 70%).

## RESULTS

**Figure 1:**
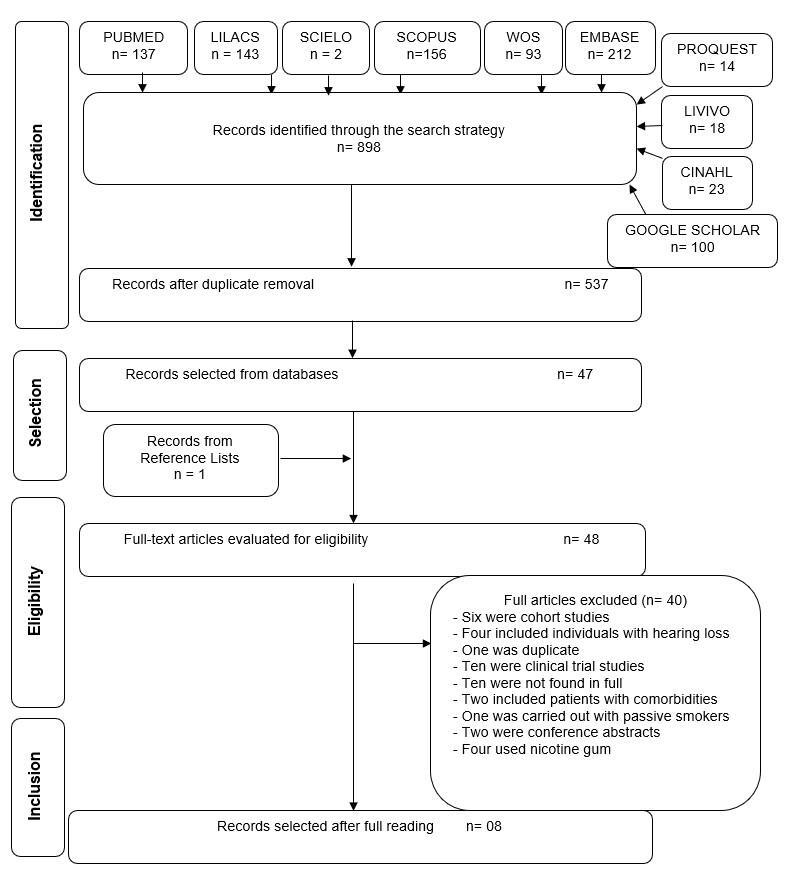
Flowchart of literature search and selection criteria

Source: Adapted from PRISMA

The first phase of the selection process resulted in 898 citations on electronic databases. After removing duplicates, a total of 537 were evaluated. After reading the titles and abstracts, 47 references were selected to be screened by full-text reading, which resulted in the inclusion of eight studies for qualitative and quantitative evaluation. A new article was added after hand searching of the reference list of articles included. Both selection and exclusion processes are shown in Figure 1.

The articles included had been published in different scientific journals (total of eight). The number of smokers included in the studies ranged from 10^19^ to 137^(19)^. Regarding the country of origin of the studies, one was from Brazil^(14)^, two were from Turkey^(20,21)^, two were from India^(7,22)^, two were from Egypt^(13,23)^, and one from Germany^(19)^. Due to the nature of the guiding question, all included studies used convenience samples. A summary of the characteristics of the eight included studies can be found in Chart 1. Regarding the test performed, three performed BAEP^(7,14,22)^, four performed P300^(19-21,23)^, and one performed Mismatch Negativity (MMN) and P300^(13)^.

**Chart 1:** Summary of studies included

**Author**	**Country**	**Age (mean in years)**	**Sex (%)**	**Sample size**	**Test used**	**Audiological outcomes**	**Conclusions**
Aşçioglu et al.^(20)^	Turkey	23 ± 2.3	NS	10 chronic smokers	P300	There were no significant differences between mean latency or amplitude values between groups (N1, P2, N2 and P300).	Chronic smoking did not interfere with the P300 response.
				10 non-smokers			
Dixit et al.^(7)^	India	Smokers 28.7 ± 2.7	100% M	30 chronic smokers	BAEP	Significant increase in the latencies of waves I and III and in the interpeak I-III, as well as a decrease in wave amplitudes in smokers.	Chronic smoking affects the auditory pathway.
		Non-smokers: 26.9 ± 1.8		30 non-smokers			
El-Shenawy et al.^(13)^	Egypt	20 min - max 50	100% M	40 smokers divided into:	MMN and P300	MMN: significant increase in wave amplitude and latency in smokers.	Smokers have a delay in processing information. Thus, chronic smoking can produce cognitive dysfunction.
				20 smokers (20 cigarettes a day)			
				20 light smokers (<20 cigarettes a day)		P300: decrease in P3 amplitude in relation to smokers, with no difference between latency values.	
				40 non-smokers			
Guney et al.^(21)^	Turkey	Smokers 40.59 years ± 13.11; non-smokers: 37.09 ± 10.01	56 smokers: 25% M (n=18); 43.75% F (n=14)	32 smokers	P300	Decreased amplitude and increased latency of N1 (in FZ) for smokers.	Chronic smoking can produce prefrontal cognitive dysfunction. Recommendation is that the P300 should be used in psychiatric patients, where the incidence of smoking is higher.
			Non-smokers: 50% M (n=16); 50% F (n=16)	32 non-smokers			
Kumar and Tandon.^(22)^	India	26.55 ± 3.36	100% M	10 smokers	BAEP	Significant increase in the absolute latency of waves I and III in smokers.	There is impairment in the conductivity of the sensory impulse along the nerve and pons in chronic smokers, due to the nicotine and toluene present in cigarettes.
				28 non-smokers			
Martins et al.^(14)^	Brazil	39.5 years	NS	20 smokers	BAEP	Significant increase in latency of wave I of the right ear and wave V for both ears and increase of interpeak III-V of both ears in smokers.	Tobacco is a risk factor for the central auditory nervous system, which can interfere with BAEP latencies and interpeaks in smokers.
				20 non-smokers			
Mostafa and Kamal.^(23)^	Egypt	34.73 ± +2.98	100% M	15 non-smokers	P300	Increased P3 latencies and lower amplitude in the group of smokers compared to non-smokers.	Chronic smoking causes decline in cognitive function and increases the electrical activity of the brain. These changes are probably caused by the toxic nicotine effect.
				30 smokers			
Neuhaus et al.^(19)^	Germany	40.7 ±14.3	48.6% M	84 smokers	P300	There was a decrease in the amplitude of the components in smokers and former smokers in relation to non-smokers. Smokers also had lower response amplitude compared to former smokers.	There is a difference in the behavior of the P300 responses between non-smokers, former smokers and smokers, pointing to a dysfunction in the generators that contribute to the recording of the components of this potential in smokers.
			51.4% F	53 former smokers			
				110 non-smokers			

**Caption:** NS = Not Specified; M = Male; F = Female; BAEP = Brainstem Auditory Evoked Potential; FZ = Front; MMN = Mismatch Negativity; min = minimum; max = maximum

In the analysis of the methodological quality of the studies included, evaluated according to the JBI Critical Appraisal Checklist for Studies Reporting Prevalence Data^(18)^, one study was classified as having a moderate risk of bias, and seven as having a low risk of bias, according to the number of responses “yes” for the eight questions in the tool adopted for quality assessment^(18)^.

The studies evaluated in this review present data that imply damage in the conductivity of the neural impulse along the auditory pathway in smokers. Of eight studies reviewed - observational studies -, one evidenced the relationship between smoking and the significant increase in the latencies of waves I and III and in the interpeaks I-III and III-V, as well as a decrease in the amplitudes of the BAEP waves^(7,14,22)^, significant increase in wave amplitude and latency of MMN in the group of smokers^(13)^, and also an increase in latencies and lower amplitude in the group of smokers in P300^(19-21,23)^.

Smoking is the main nicotine release way, as each cigarette contains about 9-13 mg nicotine, which is rapidly absorbed and transported by the bloodstream to receptors in the central nervous system^(24,25)^. Nicotinic acetylcholine receptors are widely distributed in the auditory pathways, and it is very plausible that the nicotine absorbed by smoking can influence this pathway, consequently causing conductive, mixed, sensorineural and/or central hearing loss^(24,25)^.

The degeneration of the function of the nervous system is often processed by the organism in a rostrocaudal manner. Thus, it starts with the cortex, passes through the subcortical regions, and then reaches the brainstem^(14)^. Hence the importance of investigating the effects of smoking by recording the AEP, both in the peripheral and central portions, prior to alterations in the basic audiological assessment.

When studying AEP findings in smokers, changes were found in several parameters analyzed, and the possible reason for changes in latency and amplitude of the studied components is due to the reduction in cochlear blood flow induced by nicotine^(2)^. This reduction causes changes in endocochlear potentials, cochlear microphonics and eighth nerve potentials, and nicotine central effects may be due to the alteration in the efferent neural discharge through the olivocochlear system that leads to the modulation of the response of the cochlear hair cells^(26)^.

In the studies that performed BAEP^(7,14,22)^, there was a significant increase in the absolute latency of waves I^(7,14,22)^ and III^(7,22)^ and a significant increase in the interpeaks I-III^(7)^ and III-V^(14)^. Such findings show alteration in the synchrony of the neural element in the sites that generate the response of this potential, in a diffuse manner, evidenced by the delay in the response generation.

The P300 is an endogenous auditory evoked potential, and it is identified as the result of an internal cognitive event generated in an active and voluntary manner during the performance of a specific discriminatory task between two different sound stimuli^(27,28)^. In other words, one of the stimuli is often presented, while the other occurs rarely and randomly. This vertex-positive potential and approximate latency of 300 ms appears once the individual processes a signal at a cognitive level, thus being an execution strategy of the central nervous system manifested electrophysiologically^(27,28)^.

It is known that the brain regions responsible for attention, discrimination, integration, and memory skills, such as the hippocampus, auditory cortex and frontal cortex, are the areas that stand out during the generation of the P300^(27,28)^. In order for the results to be generated, there is joint and integrate activation of the inferior parietal lobe with the medial and lateral prefrontal areas in attention processes, the heteromodal, paralimbic and hippocampus areas in memory processes, and also the auditory cortex involved in discrimination processes and linguistic auditory association^(27-29)^.

To record and analyze the results of this test, parameters such as latency and wave amplitude are considered essential^(27-29)^. Studies suggest that the more significant the frequency difference between the frequent and the rare stimuli is, the more increased the amplitude of the P300, due to the greater ease of detecting the difference between them^(27-29)^. As it is presented by four out of five articles that used this type of AEP, there was a decrease in the amplitude of response in the group of smokers^(13,19,21,23)^, and only one showed absence of significant differences between the values averages between components N1, P2, N2 and P3^(20)^. There was divergence between the observed latency relationship, since part of the studies reported increased latency^(23)^ and specifically increased N1 (in FZ) for the group of smokers^(21)^, and results were also found in which no significant difference values were obtained between latencies^(13,20)^, which shows the need for further studies with this type of potential.

The MMN test is an endogenous auditory evoked potential, which indicates responses to two stimuli, one of which is a rare stimulus and the other a frequent stimulus, which occur bilaterally in the auditory cortex^(30)^. Unlike the P300 test, the MMN does not require the patient’s attention, thus it is capable of providing information about the physiological bases for auditory discrimination without requiring the subject’s speech production^(30)^.

The MMN can be applied as a neural indicator of early auditory variations, often being used in order to observe disorders involving auditory cognition, and its main generators are found in the auditory cortex and receives contributions from the frontal cortex, thalamus, and hippocampus^(30,31)^. Pieces of Research have used the MMN to assess different aspects such as, among other factors, attention and hyperactivity disorder, detection of articulation disorders, and auditory behavior in alcohol or cigarette users^(30,31)^.

The analysis of the MMN is carried out by observing the latency and amplitude of the wave and clinical alterations can be questioned when verifying an increase in latencies or a decrease in amplitudes, where latency informs the course time of the processing activity, while the wave amplitude demonstrates the extent of neural allocation involved in cognitive processes^(31-33)^.

As previously described, nicotine triggers the activation of different receptors that can cause negative effects on the cortex, even compromising cognition^(34)^. Thus, research has been carried out with the use of the MMN to prove the effects caused by tobacco use on the auditory cortex^(34)^. One of these studies selected in this review^(13)^ pointed to a significant increase in wave amplitude and MMN latency in smokers, suggesting that smokers may present delay in information processing, just as chronic smoking can produce cognitive dysfunction.

Finally, based on the data collected and analyzed in this review, there was a predominance of alterations in the AEPs - whether BAEP, P300 or MMN - in smokers without impairment of hearing acuity. The need for more primary studies on this subject is highlighted, especially with performance of the three types of potential in the same study for a better understanding of the clinical implications observed in the same sample unit.

Some limitations can be pointed out in this systematic review, such as: small sample size; lack of studies that applied all AEPs in the same case; convenience samples in all included studies; some studies had only a single sex, and population heterogeneity. Therefore, the results should be analyzed with caution.

## CONCLUSION

The results presented by this review show that normal-hearing adult smokers present alterations in the short- and long-latency AEP tests. In the BAEP, the main altered components were the increase in the latencies of waves I and III and in the interpeaks I-III and III-V, as well as a decrease in the amplitude of the waves. In the MMN, there was a significant increase in wave amplitude and latency. In the long latency potential, P300, there was an increase in latencies and a reduction in amplitudes in components N1 (in Fz) and P3.

## References

[1] Pinto MT, Pichon-Riviere A, Bardach A (2015). The burden of smoking-related diseases in Brazil: mortality, morbidity and costs. Cad Saude Publica.

[2] Cavallieri GV, Alcarás PAS, Corazza MCA, Corazza LA (2017). The hearing of smokers: a review. Rev CEFAC.

[3] Gariepy J, Denarie N, Chironi G, Salomon J, Levenson J, Simon A (2000). Gender difference in the influence of smoking on arterial wall thickness. Atherosclerosis.

[4] Watts KL (2019). Ototoxicity: visualized in concept maps. Semin Hear.

[5] Hu H, Sasaki N, Ogasawara T, Nagahama S, Akter S, Kuwahara K (2019). Smoking, smoking cessation, and the risk of hearing loss: Japan Epidemiology Collaboration on Occupational Health Study. Nicotine Tob Res.

[6] Pezzoli M, Lofaro D, Oliva A, Orione M, Cupi D, Albera A (2017). Effects of smoking on eustachian tube and hearing. Int Tinnitus J.

[7] Dixit A, Singh YR, Mitra P, Sharma P (2020). Smoking induced alterations in auditory pathways: evidence from evoked potentials. Indian J Physiol Pharmacol.

[8] Harkrider AW, Champlin CA, McFadden D (2001). Acute effect of nicotine on non-smokers: OAEs and ABRs. Hear Res.

[9] Plourde G (2006). Auditory evoked potentials. Best Pract Res Clin Anaesthesiol.

[10] Anias CR, Limas MAMT, Kós AOA (2004). Evaluation of the influence of age in auditory brainstem response. Rev Bras Otorrinolaringol.

[11] Schochat E, Andrade AN, Takeyama FC, Oliveira JC, Sanches SGG (2009). Auditory processing: comparision between auditory middle latency response and temporal pattern tests. Rev CEFAC.

[12] Soares AJC, Sanches SGG, Neves-Lobo IF, Carvalho RMM, Matas CG, Cárnio MS (2011). Long latency auditory evoked potentials and central auditory processing in children with reading and writing alterations: preliminary data. Arq Int Otorrinolaringol.

[13] El-Shenawy AM, Hosni NA, Hamdy MM, Zirkry SS (2015). Comparison of attention and memory between smokers and non-smokers using P300 & MMN. Al-Azhar Assiut Med J.

[14] Martins DMT, Garcia CFD, Baeck HE, Frota S (2016). Brainstem auditory evoked potentials in smokers. Rev CEFAC.

[15] Page MJ, McKenzie JE, Bossuyt PM, Boutron I, Hoffmann TC, Mulrow CD (2021). The PRISMA 2020 statement: an updated guideline for reporting systematic reviews. BMJ.

[16] Canto GDL, Réus JC, Violin GC (2020). Revisões sistemáticas da literatura: guia prático.

[17] Greenhalgh T, Peacock R (2005). Effectiveness and efficiency of search methods in systematic reviews of complex evidence: audit of primary sources. BMJ.

[18] The Joanna Briggs Institute (2017). The Joanna Briggs Institute critical appraisal tools for use in JBI systematic reviews. Checklist for prevalence studies.

[19] Neuhaus A, Bajbouj M, Kienast T, Kalus P, von Haebler D, Winterer G (2006). Persistent dysfunctional frontal lobe activation in former smokers. Psychopharmacology.

[20] Aşçioglu M, Dolu N, Gölgeli A, Süer C, Özesmi Ç (2004). Effects of cigarette smoking on cognitive processing. Int J Neurosci.

[21] Guney F, Genc BO, Kutlu R, Ilhan BC (2009). Auditory P300 event-related potential in tobacco smokers. J Clin Neurosci.

[22] Kumar V, Tandon OP (1996). Brainstem auditory evoked potentials (BAEPs) in tobacco smoker. Indian J Physiol Pharmacol.

[23] Mostafa S, Kamal S (2009). Cognitive function and electroencephalogram in chronic tobacco smokers. Egypt J Neurol Psychiat Neurosurg.

[24] Veltri T, Taroyan N, Overton PG (2017). Nicotine enhances an auditory event-related potential component which is inversely related to habituation. J Psychopharmacol.

[25] Martin LF, Davalos DB, Kisley MA (2009). Nicotine enhances automatic temporal processing as measured by the mismatch negativity waveform. Nicotine Tob Res.

[26] Paschoal CP, Azevedo MF (2009). Cigarette smoking as a risk factor for auditory problems. Braz J Otorhinolaryngol.

[27] Sousa LCA, Piza MRT, Alvarenga KF, Cóser PL (2016). Eletrofisiologia da audição e emissões otoacústicas: princípios e aplicações clínicas.

[28] Didoné DD, Garcia MV, Oppitz SJ, Silva TFF, Santos SN, Bruno RS (2016). Auditory evoked potential P300 in adults: reference values. Einstein.

[29] Reis ACMB, Frizzo ACF (2015). Tratado de audiologia.

[30] Roggia SM (2015). Tratado de audiologia.

[31] Brossi AB, Borba KC, Garcia CFD, Reis ACMB, Isaac ML (2007). Verification of the Mismatch Negativity (MMN) responses in normal adult subjects. Rev Bras Otorrinolaringol.

[32] Romero ACL, Regacone SF, Lima DDB, Menezes PL, Frizzo ACF (2015). Event-related potentials in clinical research: guidelines for eliciting, recording, and quantifying Mismatch Negativity, P300, and N400. Audiol Commun Res.

[33] Ferreira DA, Bueno CD, Costa SS, Sleifer P (2017). Applicability of Mismatch Negativity in the child population: systematic literature review. Audiol Commun Res.

[34] Dani JA, Bertrand D (2007). Nicotinic acetylcholine receptors and nicotinic cholinergic mechanisms of the central nervous system. Annu Rev Pharmacol Toxicol.

