# Supplementary material for: Occurrence of changes in the auditory evoked potentials of smokers: systematic review of the literature
Source: Codas. 2023 Aug 4;35(4):e20210273. doi: 10.1590/2317-1782/20232021273en (PMC10446745; doi:10.1590/2317-1782/20232021273en)
Supplement: Translation

# Translation: Ocorrência de alterações nos potenciais evocados auditivos de fumantes: revisão sistemática da literatura

**DOI:** 10.1590/2317-1782/20232021273pt

**Authors:** Dayane Stephanie Potgurski, Georgea Espindola Ribeiro, Daniela Polo Camargo da Silva

**Affiliations:** 1 Departamento de Fonoaudiologia, Universidade Federal de Santa Catarina - UFSC - Florianópolis (SC), Brasil.; 2 Hospital das Clínicas da Faculdade de Medicina da Universidade de São Paulo - HCFMUSP - São Paulo (SP), Brasil.

**Keywords:** Fumante, Eletrofisiologia, Audição, Nervo Coclear, Potenciais Evocados Auditivos

## Abstract

**Objetivo:**

Verificar a ocorrência de alterações nos exames de potencial evocado (PEA) auditivo em adultos fumantes normo-ouvintes.

**Estratégia de pesquisa:**

Revisão sistemática da literatura de acordo com recomendações do PRISMA, buscando responder à pergunta: “Há alterações nos resultados do exame de PEA em adultos fumantes?”, estratégia PECOS. Pesquisa realizada nas bases de dados PubMed, Embase, CINAHL, LIVIVO, Scopus, Web of Science, LILACS e Scielo. Busca adicional da literatura cinzenta: Google Scholar e ProQuest e busca manual das referências dos estudos incluídos.

**Critérios de seleção:**

Foram selecionados estudos com delineamento transversal, sem restrição do ano de publicação e idioma.

**Análise dos dados:**

Primeiramente foram analisados os títulos e resumos de todos os estudos encontrados, seguido da leitura na íntegra dos estudos elegíveis.

**Resultados:**

Foram obtidos 898 artigos, que após remoção dos duplicados e análise cega por três pesquisadores, foram selecionados oito trabalhos. Grande parte dos estudos encontrou uma associação entre tabagismo ativo e alterações nos testes eletrofisiológicos.

**Conclusão:**

Adultos fumantes normo-ouvintes apresentam alterações nos exames de PEA de curta e longa latência. No potencial evocado auditivo de tronco encefálico, os principais componentes alterados foram o aumento das latências das ondas I e III e nos interpicos I - III e III - V, bem como diminuição da amplitude das ondas. No *Mismatch Negativity*, houve aumento significativo da amplitude da onda e da latência. No potencial de longa latência, P300, houve aumento das latências e redução das amplitudes nos componentes N1 (em Fz) e P3.

## INTRODUÇÃO

O consumo de tabaco é considerado como um dos fatores de risco que mais provocam a morbimortalidade em todo o mundo e sabe-se que o seu consumo é o principal meio de liberação de nicotina, que é altamente absorvida na corrente sanguínea, podendo comprometer diferentes estruturas do organismo, provocando diversos efeitos nocivos, como doenças cardíacas, acidente vascular cerebral, doença pulmonar obstrutiva crônica, além de cânceres, comprometimento cognitivo dentre outras^(1,2)^.

O tabaco também provoca diminuição da oxigenação celular, bloqueio vascular, mudanças na viscosidade sanguínea, formação de placa aterosclerótica e diminuição do aporte de oxigênio, que pode levar a um prejuízo no suprimento sanguíneo, inclusive, das vias auditivas^(1-3)^.

Sendo assim, o sistema auditivo pode sofrer influências danosas deste evento adverso e, se tratando da ototoxicidade, seus efeitos podem ser transitórios ou permanentes, a depender de quais estruturas foram acometidas ou então das características da exposição^(4)^. E também como qualquer tipo de comprometimento auditivo pode levar a uma piora da qualidade de vida do indivíduo, muitos autores têm explorado a associação entre o uso do tabaco e seu efeito na audição, ao longo dos anos^(5)^.

Um estudo recente verificou a associação prospectiva do tabagismo, a intensidade e a cessação do vício com o risco de perda auditiva, em um delineamento de coorte, por oito anos, que incluiu 50.195 participantes, com idade entre 20 e 64 anos e sem perda auditiva no início do estudo. A audiometria tonal liminar foi feita anualmente e durante o acompanhamento, 3.532 indivíduos desenvolveram perda auditiva nas altas frequências e 1.575 desenvolveram perda de audição nas baixas frequências. Concluíram que o tabagismo está associado a um risco aumentado de perda auditiva, especialmente nas altas frequências, de forma dose-resposta. O risco excessivo de perda auditiva, associado ao tabagismo, desapareceu em um período relativamente curto após a cessação do vício^(5)^.

Além da ocorrência de perda auditiva sensorioneural nas altas frequências, um estudo também observou a presença de um elevado número de fumantes com disfunção tubária, que aumentam a incidência de doenças da orelha média, por trazer sintomas inespecíficos caracterizados por plenitude auricular e dificuldade de equalização da orelha média^(6)^.

E ainda, a nicotina pode ser transportada aos receptores do sistema nervoso central podendo envolver tanto estruturas auditivas periféricas como centrais^(1,7)^. Além do mais, sabe-se que a degradação da função do sistema nervoso, em sua maioria, acontece de forma rostrocaudal, ou seja, inicia-se pelo córtex, passando pelas regiões subcorticais até atingir o tronco encefálico^(8)^. Assim, a investigação do efeito da nicotina sobre o sistema nervoso auditivo central (SNAC) tem sido também investigado através do potencial evocado auditivo (PEA).

O PEA trata-se de um conjunto de métodos que avaliam a atividade eletrobiológica, ao longo do sistema auditivo, desde orelha interna ao córtex cerebral. Dessa forma, a aplicação destes testes permite investigar condições neurofisiológicas da audição^(9)^.

Dentres os PEAs destaca-se: o potencial evocado auditivo de tronco encefálico (PEATE) considerado um potencial de curta latência, que surge em um intervalo de aproximadamente 10 ms, o mesmo permite a análise neurofisiológica da via auditiva, desde a orelha interna até o tronco encefálico alto^(10)^. O potencial evocado auditivo de média latência que surge em aproximadamente 80 ms após a estimulação, originando-se em áreas primárias do córtex auditivo, mais especificamente dos núcleos e vias auditivas existentes até no nível da região tálamo-cortical e córtex auditivo primário^(11)^, por fim o potencial evocado auditivo de longa latência que surge por volta de 100 a 700 ms após a estimulação, refletindo atividades da via auditiva nas regiões do tálamo e córtex auditivo, fornecendo informações sobre o funcionamento do sistema nervoso auditivo central (SNAC)^(12)^.

Desta forma, perdas auditivas sensorioneurais nas altas frequências de origem coclear comprometem a morfologia das ondas dos PEAs, assim como disfunções retrococleares. Neste sentido, estudos apontam que há alteração nos parâmetros de registros de exames de PEA, tais como aumento das latências e diminuição das amplitudes das respostas, em fumantes quando comparados aos não fumantes, mesmo na ausência da elevação dos limiares auditivos, sendo que esses achados podem influenciar, de alguma maneira, o correto processamento da informação acústica^(7,13,14)^.

É provável que diversos sítios anatômicos, responsáveis pela produção da atividade neuroelétrica da via auditiva, em resposta a uma estimulação acústica, se comporte de forma diferente em fumantes, o que torna necessário avaliar quais são as evidências disponíveis na literatura que comprovem a existência ou não dessa associação.

Diante do exposto acima, estudos têm apontado a necessidade de se realizar testes complementares a avaliação audiológica básica a fim de se investigar a extensão da lesão ocasionada pela exposição constante ao tabaco e auxiliar no melhor entendimento das alterações encontradas, além de detalhar os tipos de alterações que o fumo ocasiona ao SNAC^(2,7,13,14)^.

Assim, a investigação dos PEAs se mostra útil no diagnóstico diferencial da perda auditiva sensorioneural trazendo informações importantes que podem objetivamente indicar se a lesão está situada em nível coclear e/ou retrococlear, bem como mostrar alteração precoce nos sítios geradores da resposta neural, antes de se detectar mudanças na avaliação audiológica básica^(2,7,13,14)^.

## OBJETIVO

Desse modo, o objetivo do presente estudo foi verificar a ocorrência de alterações nos exames de PEA em adultos fumantes normo-ouvintes, por meio de uma revisão sistemática da literatura.

### Estratégia de pesquisa

Esta revisão sistemática seguiu as recomendações do *Preferred Reporting Items for Systematic Reviews and Meta-analyses* - PRISMA^(15)^.

Foram considerados elegíveis para essa revisão sistemática estudos observacionais realizados em adultos (idade maior ou igual a 18 anos e menores de 60 anos) tabagistas, que tiveram como objetivo avaliar a integridade da via auditiva por meio do exame de PEA. Não houve restrição do tempo de publicação dos estudos. Foram excluídos os seguintes estudos: (1) artigos de revisão, cartas, estudos de caso e resumos para eventos; (2) estudos realizados em fumantes associados com outras doenças; (3) estudos que incluíram indivíduos com idade inferior a 18 anos ou maiores de 60 anos; (4) estudos realizados em fumantes com alterações auditivas prévias ao vício; (5) estudos sem grupo controle; (6) estudos que não tinham a versão na íntegra disponível.

Para a condução do estudo foi utilizada a seguinte pergunta norteadora: “Há alterações nos resultados do exame de potenciais evocados auditivos em adultos fumantes?”. Esta pergunta foi organizada no formato PECOS^(16)^ (*Patient, Exposure, Comparison, Outcomes, Studies*). Assim, o acrônimo (P) consiste em população (adultos), (E) consiste na exposição ao tabaco, (O) consiste em qualquer alteração nos exames de potenciais evocados auditivos, (C) adultos sem exposição ao tabaco e (S) consiste no desenho de estudos incluídos, observacionais - transversais.

Foram desenvolvidas estratégias de busca eletrônica individual, sendo utilizada a combinação dos seguintes descritores em português e em inglês, respectivamente: “Fumantes”, “Potenciais Evocados Auditivos”, “Eletrofisiologia”, “Nervo Coclear”, “*Smokers*”, “*Auditory Evoked Potentials*”, “*Electrophysiology*”, “*Cochlear Nerve*”. Para contemplar os determinados eixos temáticos, foram utilizados os operadores booleanos “OR” e “AND”, de acordo com MESH/DECS, para cada uma das seguintes bases de dados: PubMed, Embase, CINAHL, LIVIVO, Scopus, Web of Science, LILACS, Scielo. Uma pesquisa adicional da literatura cinzenta foi realizada acessando o Google Scholar e ProQuest. Além disso, foram realizadas buscas manuais das referências dos estudos incluídos, conforme recomendado por Greenhalgh e Peacock^(17)^. Um *software* gerenciador de referência (EndNote Web®) foi usado para coletar as referências e excluir as duplicatas. A data de coleta nas bases foi realizada no dia 24 de março de 2021. Foram selecionados os estudos que responderam a pergunta de pesquisa, sem restrição da data de publicação e idioma.

### Critérios de seleção

A etapa de seleção foi realizada em duas fases. Na fase um, os títulos e resumos de todas as citações do banco de dados identificadas foram selecionados de forma independente por dois revisores. Os estudos que não preencheram os critérios de elegibilidade foram excluídos. Na fase dois, os mesmos dois revisores aplicaram os critérios de elegibilidade ao texto completo dos estudos. Um terceiro revisor foi consultado em caso de desacordo que não tenha sido resolvido por uma discussão de consenso entre os dois revisores.

### Análise dos dados

Dois autores coletaram as informações necessárias dos estudos selecionados. Um terceiro autor confirmou a veracidade das informações coletadas por meio da conferência do texto completo dos artigos com as informações selecionadas pelos dois primeiros autores. Quaisquer controvérsias, neste processo, foram discutidas e estabelecido um consenso. Os dados extraídos dos estudos foram: características dos estudos (autores, ano de publicação, país, tipo de estudo), características da população (tamanho da amostra, faixa etária do grupo estudado), característica de exposição (caracterização do tabagismo) e características do desfecho (tipo de alteração encontrada na via auditiva retrococlear, tipo de PEA utilizado e principais achados apresentadas pelos estudos).

A avaliação do risco de viés dos estudos selecionados foi avaliada pelo *JBI Critical Appraisal Checklist for Studies Reporting Prevalence Data* ^(18)^. O primeiro e o segundo autores realizaram esta avaliação de forma independente. Quaisquer discordâncias surgidas foram resolvidas com a ajuda do terceiro autor.

Para cada domínio da ferramenta foi atribuída uma das seguintes respostas: “*Yes*”, “*No*”, “*Unclear*” ou “*Not Applicable*”^(13)^. Para a porcentagem de “Yes” de cada estudo analisado, foi considerado com risco alto de viés (> 49%) ou moderado (50 a 69%) ou baixo (> 70%).

## RESULTADOS

A primeira fase do processo de seleção resultou em 898 citações nas bases de dados eletrônicas. Após a remoção de duplicados, um total de 537 foi avaliado. Após a leitura de títulos e resumos, 47 referências foram selecionadas para serem triadas por meio da leitura do texto na íntegra, o que resultou na inclusão de oito estudos para avaliação qualitativa e quantitativa. Foi adicionado um artigo novo após a pesquisa na lista de referências dos artigos incluídos. Tanto a seleção quanto os processos de exclusão são apresentados na Figura 1.

**Figura 1:** Fluxograma de pesquisa da literatura e critérios de seleção

Fonte: Adaptado do PRISMA

Os artigos incluídos foram publicados em diferentes revistas científicas (total de oito). O número de fumantes incluídos nos estudos variou de 10^19^ a 137^(19)^. Em relação ao país de origem dos estudos, um foi do Brasil^(14)^, dois foram da Turquia^(20,21)^, dois foram da Índia^(7,22)^, dois foram do Egito^(13,23)^ e um da Alemanha^(19)^. Devido a natureza da pergunta norteadora, todos os estudos incluídos usaram amostras de conveniência. A síntese das características dos oito estudos incluídos pode ser encontrada no Quadro 1. Em relação ao exame realizado, três executaram o PEATE^(7,14,22)^, quatro realizaram o P300^(19-21,23)^ e um o MMN e o P300^(13)^.

**Quadro 1:** Síntese dos estudos incluídos

**Autor**	**País**	**Idade (média em anos)**	**Sexo (%)**	**Tamanho da amostra**	**Exame Utilizado**	**Desfechos audiológicos**	**Conclusões**
Aşçioglu et al.^(20)^	Turquia	23 ± 2,3	NE	10 fumantes crônicos	P300	Não houve diferenças significativas entre os valores médios de latência ou amplitude entre os grupos (N1, P2, N2 e P300).	O tabagismo crônico não interferiu na resposta do P300.
				10 não fumantes			
Dixit et al.^(7)^	Índia	Fumantes: 28,7 ± 2,7	100% M	30 fumantes crônicos	PEATE	Aumento significativo das latências das ondas I e III e no interpico I - III, bem como diminuição das amplitudes das ondas nos participantes fumantes.	O tabagismo crônico afeta a via auditiva.
		Não fumantes: 26,9 ± 1,8		30 não fumantes			
El-Shenawy et al.^(13)^	Egito	20 mín - máx 50	100% M	40 fumantes subdivididos em:	MMN e P300	MMN: aumento significativo da amplitude da onda e da latência no grupo de fumantes.	Fumantes apresentam atraso no processamento da informação. Assim, o tabagismo crônico pode produzir disfunção cognitiva.
				20 fumantes (20 cigarros por dia)			
				20 fumantes leves (< 20 cigarros por dia)		P300: diminuição da amplitude de P3 em relação aos fumantes, sem diferença entre os valores das latências.	
				40 não fumantes			
Guney et al.^(21)^	Turquia	Fumantes: 40,59 anos ± 13,11; Não fumantes: 37,09 anos ± 10,01	56 Fumantes: 25% M (n=18); 43,75% F (n=14)	32 fumantes	P300	Diminuição da amplitude e aumento da latência de N1 (em FZ) para o grupo de fumantes.	O tabagismo crônico pode produzir disfunção cognitiva pré-frontal. Recomendam o uso do P300 em pacientes psiquiátricos, onde a incidência do tabagismo é maior.
			Não fumantes: 50% M (n=16) 50% F (n=16)	32 não fumantes			
Kumar e Tandon.^(22)^	Índia	26,55 ± 3,36	100% M	10 Fumantes	PEATE	Aumento significativo da latência absoluta das ondas I e III nos fumantes.	Há prejuízo na condutividade do impulso sensorial ao longo do nervo e ponte em fumantes crônicos, em decorrência da nicotina e tolueno presentes no cigarro.
				28 não fumantes			
Martins et al.^(14)^	Brasil	39,5 anos	NE	20 fumantes	PEATE	Aumento significativo da latência da onda I da orelha direita e da onda V para ambas as orelhas e aumento do interpico III - V de ambas as orelhas em tabagistas	Tabaco é um fator de risco para o sistema nervoso auditivo central, que pode interferir nas latências e interpicos do PEATEde tabagistas.
				20 não fumantes			
Mostafa e Kamal.^(23)^	Egito	34,73 ± 2,98	100% M	15 não fumantes	P300	Aumento das latências e amplitude menores de P3 no grupo de fumantes em relação aos não fumantes.	O tabagismo crônico causa declínio da função cognitiva, aumenta a atividade elétrica do cérebro. Essas mudanças provavelmente resultam do efeito tóxico da nicotina.
				30 fumantes			
Neuhaus et al.^(19)^	Alemanha	40,7 ± 14,3	48,6% M	84 fumantes	P300	Houve diminuição da amplitude dos componentes no grupo de fumantes e ex-fumantes em relação aos não fumantes. Os fumantes também apresentaram menor amplitude de resposta em relação aos ex-fumantes.	Há diferença no comportamento das respostas do P300 entre não fumantes, ex fumantes e fumantes, apontando uma disfunção nos geradores que contribuem para o registro dos componentes desse potencial nos indivíduos fumantes.
			51,4% F	53 ex-fumantes			
				110 não fumantes			

**Legenda:** NE = Não Especificado; M = Sexo Masculino; F = Feminino; PEATE = Potencial Evocado Auditovo de Tronco Encefálico; FZ = Frontal; MMN = Mismatch Negativity; mín = mínimo; máx = máximo

Na análise da qualidade metodológica dos estudos incluídos, avaliado de acordo com *JBI Critical Appraisal Checklist for Studies Reporting Prevalence Data* ^(18)^, um estudo foi classificado como tendo moderado risco de viés, e sete com baixo risco de viés, de acordo com o número de respostas “sim” para as oito perguntas na ferramenta adotada para a avaliação da qualidade^(18)^.

Os estudos avaliados nesta revisão mostram dados que implicam em prejuízos na condutividade do impulso neural ao longo da via auditiva em indivíduos fumantes. A partir de oito trabalhos revisados, sendo estes com tipo de estudo observacional, evidenciou-se a relação entre o tabagismo com o aumento significativo das latências das ondas I e III e nos interpicos I - III e III - V, bem como diminuição das amplitudes das ondas do potencial evocado auditivo de tronco encefálico (PEATE)^(7,14,22)^, aumento significativo da amplitude da onda e da latência do Mismatch Negativity (MMN) no grupo de fumantes^(13)^ e ainda, aumento das latências e amplitude menores no grupo de fumantes no exame P300^(19-21,23)^.

O tabagismo é o principal sistema de liberação da nicotina, pois cada cigarro contém cerca de 9-13 mg de nicotina que é rapidamente absorvida e transportada pela corrente sanguínea aos receptores no sistema nervoso central^(24,25)^. Os receptores nicotínicos de acetilcolina têm ampla distribuição nas vias auditivas, o que é muito provável que a nicotina absorvida pelo fumo possa exercer influência nesta via, e consequentemente provocar perdas auditivas de natureza condutiva, mista, sensorioneural e/ou central^(24,25)^.

A deterioração da função do sistema nervoso central muitas vezes é processada pelo organismo de forma rostro-caudal. Sendo assim, inicia-se pelo córtex, passa pelas regiões subcorticais quando então atinge o tronco encefálico^(14)^. Daí a importância da investigação dos efeitos do tabagismo por meio do registro dos PEA, tanto na porção periférica como central, previamente a alterações na avaliação audiológica básica.

Ao estudar os achados dos PEA em fumantes encontram-se mudanças em diversos parâmetros analisados e a possível razão para as alterações na latência e nas amplitudes dos componentes estudados se deve ao fato da redução do fluxo sanguíneo coclear induzida pela nicotina^(2)^. Esta redução causa mudanças nos potenciais endococleares, no microfonismo coclear e nos potenciais do oitavo nervo e é possível também que os efeitos centrais da nicotina são em decorrência da alteração na descarga neural eferente através da sistema olivococlear que leva à modulação da resposta das células ciliadas da cóclea^(26)^.

Para os estudos que realizaram o PEATE^(7,14,22)^, observou-se aumento significativo da latência absoluta das ondas I^(7,14,22)^ e III^(7,22)^ e aumento significativo do interpico I - III^(7)^ e III - V^(14)^. Tais achados mostram alteração na sincronia do elemento neural nos sítios geradores da resposta deste potencial, de forma difusa, evidenciado pelo atraso na formação da resposta.

O P300 é um potencial evocado auditivo endógeno, sendo designado como o resultado de um evento cognitivo interno gerado de forma ativa e voluntária durante o desempenho de uma tarefa discriminatória específica entre dois estímulos sonoros diferentes entre si^(27,28)^. Ou seja, um dos estímulos é apresentado de forma frequente, enquanto que o outro ocorre raramente e de forma aleatória. Este potencial de vértex-positivo e latência aproximada de 300 ms aparece uma vez que o indivíduo processe um sinal em nível cognitivo, sendo assim uma estratégia de execução do sistema nervoso central manifestada eletrofisiologicamente^(27,28)^.

Sabe-se que as regiões cerebrais responsáveis pelas habilidades de atenção, discriminação, integração e memória, como por exemplo o hipocampo, córtex auditivo e o córtex frontal, são as áreas que se sobressaem durante a geração do P300^(27,28)^. Para que os resultados sejam gerados, são ativadas de forma conjunta e integrada o lobo parietal inferior com áreas pré frontais medial e lateral nos processos de atenção, das áreas paralímbicas heteromodais e do hipocampo nos processos de memória e, ainda, o córtex auditivo envolvidos nos processos de discriminação e associação auditivo linguística^(27-29)^.

Para registro e análise dos resultados deste teste, parâmetros como latência e amplitude de onda são considerados fundamentais^(27-29)^. Estudos sugerem que a amplitude do P300 aumenta quanto mais significativa for a diferença de frequência entre o estímulo frequente e o raro, devido a maior facilidade de detecção da diferença entre eles^(27-29)^. As pesquisas selecionadas evidenciam que se tratando de sujeitos fumantes, quatro dos cinco artigos que utilizaram este tipo de PEA, houve diminuição da amplitude de resposta no grupo de fumantes^(13,19,21,23)^, apenas um deles evidenciou ausência de diferenças significativas entre os valores médios entre os componentes N1, P2, N2 e P3^(20)^. Houve divergência entre a relação das latências observadas, uma vez que parte dos estudos referiu aumento da latência^(23)^ e especificamente aumento de N1 (em FZ) para o grupo de fumantes^(21)^, também foram encontrados resultados em que não foram obtidos valores de diferença significativa entre as latências^(13,20)^, o que mostra a necessidade de mais estudos com este tipo de potencial.

O teste Mismatch Negativity (MMN) é um potencial evocado auditivo endógeno, que indica respostas de dois estímulos, sendo um deles o estímulo raro e o outro, um estímulo frequente, os quais ocorrem bilateralmente no córtex auditivo^(30)^. Diferente do teste P300, o MMN não requer a atenção do paciente, sendo assim capaz de fornecer informações sobre as bases fisiológicoas para a discriminação auditiva, sem requerer a produção da fala do sujeito^(30)^.

O MMN pode ser aplicado como um indicador neural de variações auditivas precoces, sendo muitas vezes, aplicado a fim de se observar transtornos envolvendo a cognição auditiva, e seus principais geradores encontram-se no córtex auditivo e recebe contribuições do córtex frontal, tálamo e hipocampo^(30,31)^. Pesquisas têm utilizado o MMN para avaliar diferentes aspectos como: transtorno de atenção e hiperatividade, detecção de distúrbios articulatórios, comportamento auditivo em usuários de álcool ou cigarro entre outros fatores^(30,31)^.

A análise do MMN é realizada através da observação da latência e amplitude da onda e ao se verificar aumento das latências ou diminuição das amplitudes, alterações clínicas podem ser questionadas, sendo que a latência informa o tempo de curso da atividade do processamento, enquanto que a amplitude da onda demonstra a extensão da alocação neural envolvida nos processos cognitivos^(31-33)^.

Como descrito anteriormente, a nicotina provoca a ativação de diferentes receptores que podem ocasionar efeitos negativos ao córtex comprometendo até mesmo a cognição^(34)^. Dessa forma, pesquisas têm sido feitas com o uso do MMN para comprovar os efeitos provocados pelo uso do tabaco no córtex auditivo^(34)^. Um desses estudos selecionados na presente revisão^(13)^, apontou aumento significativo da amplitude da onda e da latência do MMN nos fumantes, sugerindo que sujeitos fumantes possam apresentar atraso no processamento da informação, assim como o tabagismo crônico pode produzir disfunção cognitiva.

Por fim, a partir dos dados coletados e analisados nesta revisão, houve predomínio de alterações em qualquer um dos PEA, seja PEATE, P300 ou MMN, em indivíduos fumantes sem prejuízo da acuidade auditiva. Destaca-se a necessidade de mais estudos primários sobre este assunto, principalmente na realização dos três tipos de potencial num mesmo estudo para melhor entendimento das implicações clínicas observadas numa mesma unidade amostral.

Algumas limitações podem ser apontadas nesta revisão sistemática, como o pequeno número da amostra dos estudos incluídos, não foi encontrado estudos que aplicassem todos os PEAs na mesma casuística, todos os estudos incluídos foram conduzidos com amostras de conveniência, alguns eram restritos a um único sexo, e a heterogeneidade da população deve ser considerada, portanto, os resultados devem ser analisados com cautela.

## CONCLUSÃO

Os resultados apresentados por esta pesquisa evidenciam que adultos fumantes normo-ouvintes apresentam alterações nos exames de PEA de curta e longa latência. No PEATE, os principais componentes alterados foram o aumento das latências das ondas I e III e nos interpicos I - III e III - V, bem como diminuição da amplitude das ondas. No MMN, houve aumento significativo da amplitude da onda e da latência. No potencial de longa latência, P300, houve aumento das latências e redução das amplitudes nos componentes N1 (em Fz) e P3.
